# GeneXpert MTB/RIF Outperforms Mycobacterial Culture in Detecting* Mycobacterium tuberculosis* from Salivary Sputum

**DOI:** 10.1155/2018/1514381

**Published:** 2018-04-01

**Authors:** Jin Shi, Wenzhu Dong, Yifeng Ma, Qian Liang, Yuanyuan Shang, Fen Wang, Hairong Huang, Yu Pang

**Affiliations:** ^1^National Clinical Laboratory on Tuberculosis, Beijing Key Laboratory on Drug-Resistant Tuberculosis Research, Beijing Chest Hospital, Capital Medical University, Beijing Tuberculosis and Thoracic Tumor Institute, Beijing, China; ^2^Beijing Key Laboratory for Pediatric Diseases of Otolaryngology, Head and Neck Surgery, MOE Key Laboratory of Major Diseases in Children, Beijing Pediatric Research Institute, Beijing Children's Hospital, Capital Medical University, National Center for Children's Health, Beijing 100045, China; ^3^Inner Mongolia Medical University, Hohhot 010059, China

## Abstract

GeneXpert MTB/RIF (Xpert) assay has been endorsed for the diagnosis of pulmonary TB due to its high sensitivity and specificity for culture positive TB. There is no doubt that Xpert could not be more sensitive than mycobacterial culture, while the positive rate of Xpert among sputum samples was higher than that of mycobacterial culture in our laboratory. We therefore carried out a prospective study to determine a potential explanation for this unexpected result regarding the clinical use of Xpert. Overall, a total of 558 patients meeting inclusion criteria were enrolled in final analysis between August 2017 and September 2017 in Beijing Chest Hospital. The overall positive rate of Xpert among sputum samples was 45.9% (256/558), which was significantly higher than that of liquid culture (33.4%, 184/558; *P* < 0.01). The percentage of culture negative result in salivary sputum was significantly higher than that in mucoid sputum [odds ratio (OR): 5.04, 95% confidence interval (95% CI): 2.74–9.28; *P* < 0.01]. In addition, the TB cases having previous treatment history had a higher proportion of culture negative result than new cases (OR: 4.26, 95% CI: 1.61–11.28; *P* = 0.01). In conclusion, the results of this study demonstrate that Xpert outperforms mycobacterial culture in detecting MTB from salivary sputum. In addition, the previously treated patients are more likely to yield negative culture results. Our data will provide important hints to formulate an appropriate diagnostic algorithm for pulmonary tuberculosis based on the appearance of sputum samples.

## 1. Introduction

Tuberculosis, caused by* Mycobacterium tuberculosis* complex (MTBC), is now the leading cause of morbidity and mortality from an infectious disease worldwide, with an estimated 10.4 million new cases and 1.3 million deaths in 2016 (WHO, 2017). China has the third largest burden of tuberculosis in the world, accounting for 8.6% of global tuberculosis incidence (WHO, 2017). Although China has achieved great progress in TB control by the implementation of effective TB control strategy, the low detection rate of bacteria-positive TB cases poses a new challenge that hampers efforts at tuberculosis control [1]. According to the analysis of nationwide data, only 30% of the reported TB patients had positive laboratory evidence in this country, which is significantly lower than the global average rate of 50% (WHO, 2017). Hence, this unsatisfactory situation highlights the urgent need to develop and employ higher sensitivity assays for identifying TB patients.

The World Health Organization (WHO) has endorsed GeneXpert MTB/RIF (Xpert) assay (Cepheid, CA, USA) for the diagnosis of pulmonary TB due to its high sensitivity and specificity for culture positive TB (WHO, 2013). In addition, several recent analyses suggest that Xpert yields promising efficacy in detecting extrapulmonary TB (EPTB), which facilitates the formulation of WHO guidelines for the application of Xpert in EPTB diagnosis [2, 3]. Despite the impressive performance of Xpert from numerous clinical trials, there is no doubt that Xpert could not be more sensitive than mycobacterial culture, the gold standard for TB diagnosis [4]. To our surprise, the positive rate of Xpert among sputum samples was higher than that of mycobacterial culture in our laboratory. We therefore carried out a prospective study to determine a potential explanation for this unexpected result regarding the clinical use of Xpert.

## 2. Methods

### 2.1. Study Population

From August 2017 through September 2017, we performed a prospective observational study to explore the potential reasons for the high detection rate of Xpert compared with that of liquid culture. All the patients with presumptive pulmonary TB admitted to Beijing Chest Hospital were consecutively enrolled in this study. The patients who had received >2 weeks of anti-TB treatment within the last 2 months were excluded from this study. Each patient was required to collect a minimum volume of 3.0 mL sputum for further examination. The demographic characteristics and treatment history of recruited patients were obtained by reviewing the medical records.

### 2.2. Laboratory Examination

The hospital clinical laboratory performed microbiologic and Xpert testing following standard protocols. Upon the receipt of sputum, the sputum appearance was classified as salivary, mucoid, bloody, and caseous. All sputum samples were detected with fluorescent smear microscopy and scored as negative, scanty, 1+, 2+, 3+, 4+ according to the national guidelines for smear microscopy [5]. In addition, 1 mL of sputum was digested with N-acetyl-L-cysteine- (NALC-) sodium hydroxide (NaOH) (final NaOH concentration of 1.5%) for 15 min. Following the neutralization with autoclaved phosphate buffer solution (PBS, pH = 6.8), the concentrated sediment was resuspended with PBS. Then 0.5 mL of sediment suspension was inoculated into a MGIT tube supplemented with OADC and PANTA. In addition, 1 mL of sputum was mixed with 2 mL of Xpert sample reagent and incubated for 15 min at room temperature with intermittent shaking. After processing, 2 mL of mixed sample was transferred to the Xpert cartridge, and the reports were automatically generated after 90 min of amplification. The bacterial loads of sputum specimens were divided into four levels by the Xpert reports, including high, medium, low, and very low levels [6].

### 2.3. Statistical Analysis

The Pearson chi-square test was performed to compare the positive rates between Xpert and liquid culture and also analyze the distribution of categorical variables between Xpert positive/culture negative and Xpert positive/culture positive groups. The difference was declared as statistically significant if *P* value was less than 0.05. All the statistical analyses were performed using SPSS 15.0 (SPSS Inc., Chicago, IL, USA).

## 3. Results

**Figure 1:**
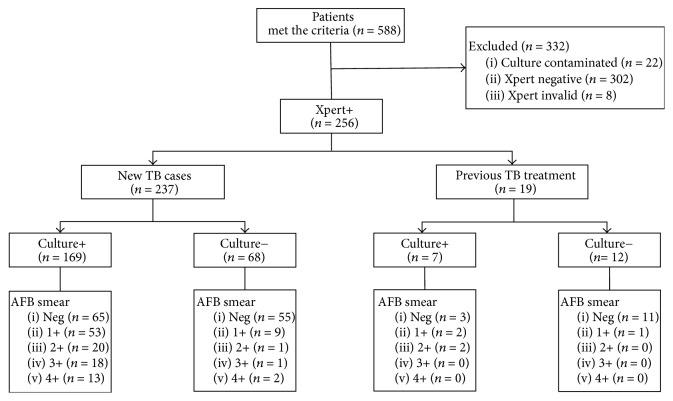
Enrollment of participants in this study.

Overall, a total of 588 patients meeting inclusion criteria were entered in the study between August 2017 and September 2017. The sputum samples underwent diagnostic testing, including smear microscopy, liquid culture, and Xpert examination, respectively. Of 588 cases, 30 were excluded due to culture contamination (*n* = 22) and failed Xpert results (*n* = 8) so that 558 were included in the final analysis (Figure 1).

The overall positive rates of Xpert and liquid culture among sputum samples were 45.9% (256/558) and 33.4% (184/558), respectively. Statistical analysis revealed that the positive rate of Xpert was significantly higher than that of liquid culture (*P* < 0.01). Out of 558 cases, 176 (31.5%) were identified as Xpert positive/culture positive, 80 (14.3%) as Xpert positive/culture negative, 8 (1.4%) as Xpert negative/culture positive, and 294 (52.7%) as Xpert negative/culture negative.

We further analyzed the factor associated with Xpert positive/culture negative results. As shown in Table 1, the percentage of culture negative result in salivary sputum was significantly higher than that in mucoid sputum (OR: 5.04, 95% CI: 2.74–9.28; *P* < 0.01), whereas there was no significant difference between other groups (*P* > 0.05). Statistical analysis revealed that the TB cases having previous treatment history had a higher proportion of culture negative result than new cases (OR: 4.26, 95% CI: 1.61–11.28; *P* = 0.01). Compared with sputum samples graded 1+, we found that the negative sputum samples were more likely to yield Xpert positive/culture negative results (OR: 5.34, 95% CI: 2.51–11.35; *P* < 0.01).

## 4. Discussion

Early diagnosis of tuberculosis is essential for initiating an effective treatment regimen and preventing its transmission in the community [7]. Recent advances in diagnostic technologies emphasize the role of molecular diagnostics for detecting bacteria from clinical specimens with acceptable turnaround time [8]. In this study, we observed that Xpert outperformed mycobacterial culture in the detection of MTB from salivary sputum. Consistent with our report, a recent study from Acuña-Villaorduña and colleagues has demonstrated that the discordant culture negative/Xpert positive results are associated with mucoid/salivary sputum [9], and our results further confirm that these inconsistencies are majorly contributed to salivary sputum rather than mucoid sputum. Therefore the high proportion of salivary sputum among all sputum samples detected in this study may be important for explaining the increased detection rate of MTB using Xpert compared with mycobacterial culture. As has been demonstrated previously, the limit of detection of mycobacterial culture is as low as 10 to 100 CFU/mL, which is not inferior to GeneXpert [4]. It is interesting to explore the potential reason for such extraordinary situation. Due to having a thick cell lipid-enriched wall, the MTB buoyant densities ranged from 0.79 to 1.07 g/cm^3^, no higher than that of clinical samples [10]. Given that the buoyant density is essential for effective sedimentation or centrifugation, we hypothesize that the concentration of salivary specimens by centrifugation may yield unsatisfactory efficacy in the recovery of mycobacteria, whereas the instinct viscosity and residual debris of sputum improve the recovery rate of mycobacteria, thereby leading to the decrease in the reported positive rate from salivary sputum. Our results echo previous findings that sputum quality exhibits a strong association with the presence or absence of MTB in sputum tested with smear and culture except for Xpert [9, 11]. In view of these data, Xpert MTB/RIF provides a superior close-to-patient test for identifying MTB from salivary sputum in comparison to conventional bacterial methods.

Another notable finding of this study is that we identified that the previously treated patients were more likely to yield negative culture results. Mycobacterial culture relies on the viable microorganisms in the sputum samples. The prior exposure to anti-TB drug among previously treated patients may reduce the density of tubercle bacillus and also weaken the vitality of surviving bacteria in the sputum samples [12]. Despite being undetected by culture methods, the dead or weakened tubercle bacilli would be identified by molecular diagnostics, which may appear to be important factor affecting the lower recovery rate by liquid culture [13]. In addition, dormant bacteria represent bacteria in a state of dormancy, which remain viable but do not form colonies directly on culture media [14]. There is strong evidence that the history of previous treatments against tuberculosis is an effective condition to induce the formulation of dormant bacteria [14]. Hence, we hypothesize that the presence of these special bacteria's population in the previously treated patients may affect the detection rate of mycobacterial culture. Further experimental research is required to investigate the contribution of dormant bacteria in decreasing the recovery rate of tubercle bacilli from sputum samples collected from previously treated TB patients.

In conclusion, the results of this study demonstrate that Xpert outperforms mycobacterial culture in detecting MTB from salivary sputum. In addition, the previously treated patients are more likely to yield negative culture results. Given the important role of Xpert and mycobacterial culture in detecting TB, our data will provide important hints to formulate an appropriate diagnostic algorithm for pulmonary tuberculosis based on the appearance of sputum samples.

## Figures and Tables

**Table 1:** Factors associated with negative culture results among 256 pulmonary TB patients having positive Xpert results in this study.

Characteristic	Number of patients with positive Xpert (%)		OR (95% CI)	*P* value
	Culture positive (*n* = 176)	Culture negative (*n* = 80)		
*Gender*				
Male	105 (59.66)	49 (61.25)	1.07 (0.62–1.83)	0.81
Female	71 (40.34)	31 (38.75)	Ref.	-
*Age group (years)*				
<25	26 (14.77)	16 (20.00)	1.71 (0.77–3.76)	0.18
25–44	61 (34.66)	22 (27.50)	Ref.	-
45–64	41 (23.30)	21 (26.25)	1.42 (0.69–2.91)	0.34
≥64	48 (27.27)	21 (26.25)	1.21 (0.60–2.46)	0.60
*Treatment history*				
New case	169 (96.02)	68 (85.00)	Ref.	-
Retreated	7 (4.98)	12 (15.00)	4.26 (1.61–11.28)	0.01
*Sputum appearance*				
Salivary	67 (38.07)	59 (73.75)	5.04 (2.74–9.28)	<0.01
Mucoid	103 (58.53)	18 (22.50)	Ref.	-
Bloody	3 (1.70)	2 (2.50)	3.82 (0.60–24.46)	0.18
Caseous	3 (1.70)	1 (1.25)	1.91 (0.19–19.37)	0.50
*AFB smear*				
Negative	68 (38.64)	66 (82.5)	5.34 (2.51–11.35)	<0.01
1+	55 (31.25)	10 (12.5)	Ref.	-
2+	22 (12.5)	1 (1.25)	0.25 (0.03–2.07)	0.17
3+	18 (10.23)	1 (1.25)	0.31 (0.04–2.55)	0.25
4+	13 (7.39)	2 (2.50)	0.85 (0.17–4.34)	1.00
*Bacterial load*				
Very low	18 (10.23)	27 (33.75)	10.50 (4.57–24.15)	<0.01
Low	41 (23.30)	39 (48.75)	6.66 (3.22–13.79)	<0.01
Medium	91 (51.70)	13 (16.25)	Ref.	
High	26 (14.77)	1 (1.25)	0.27 (0.03–2.16)	0.30
